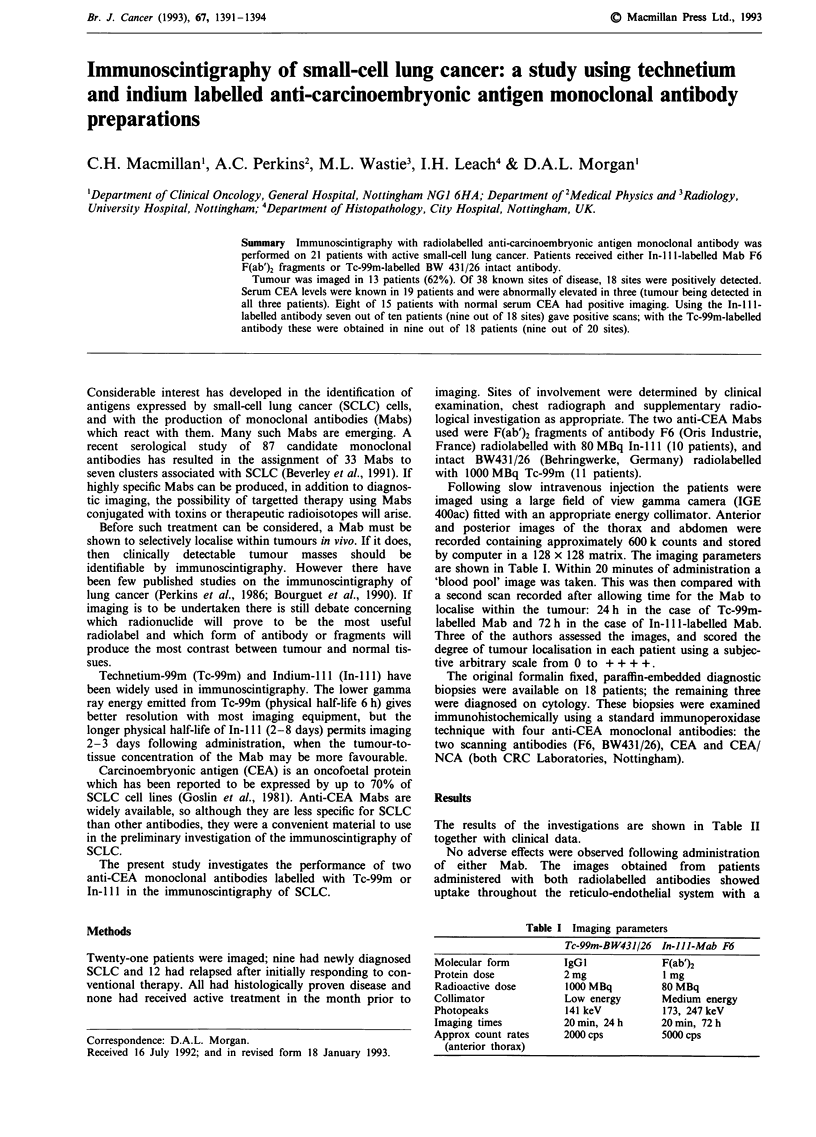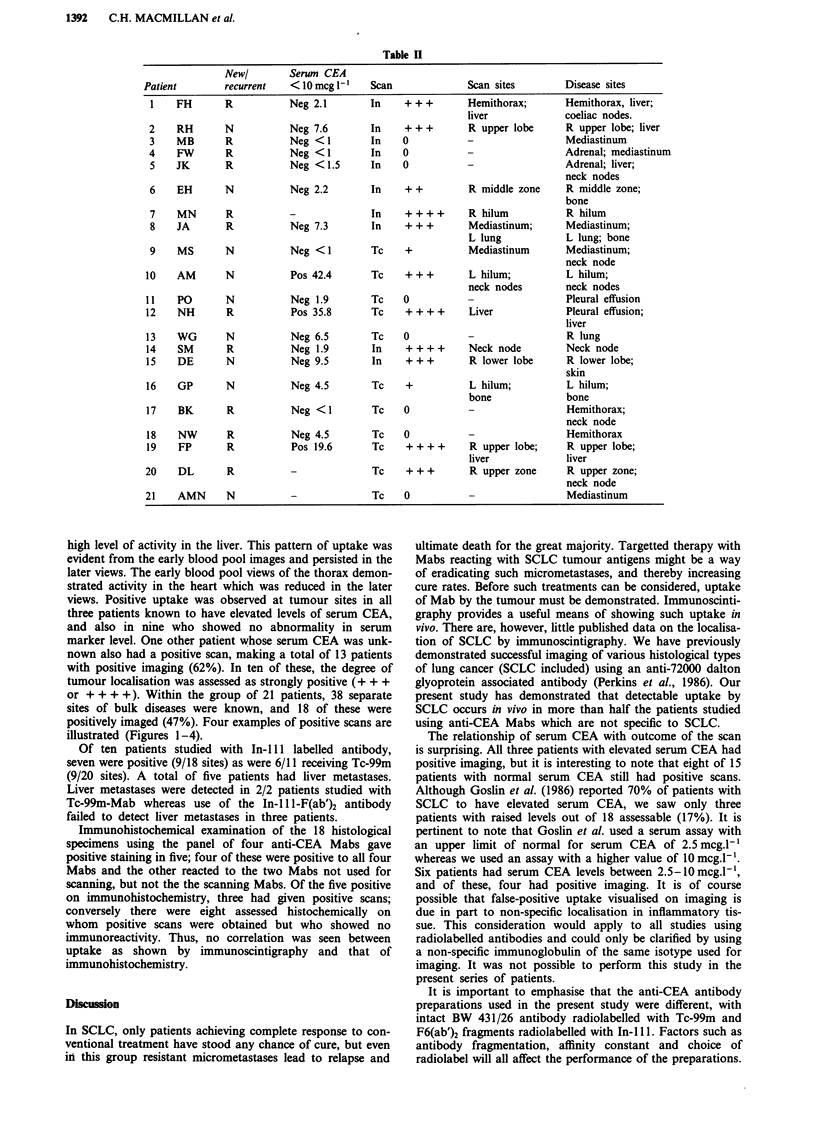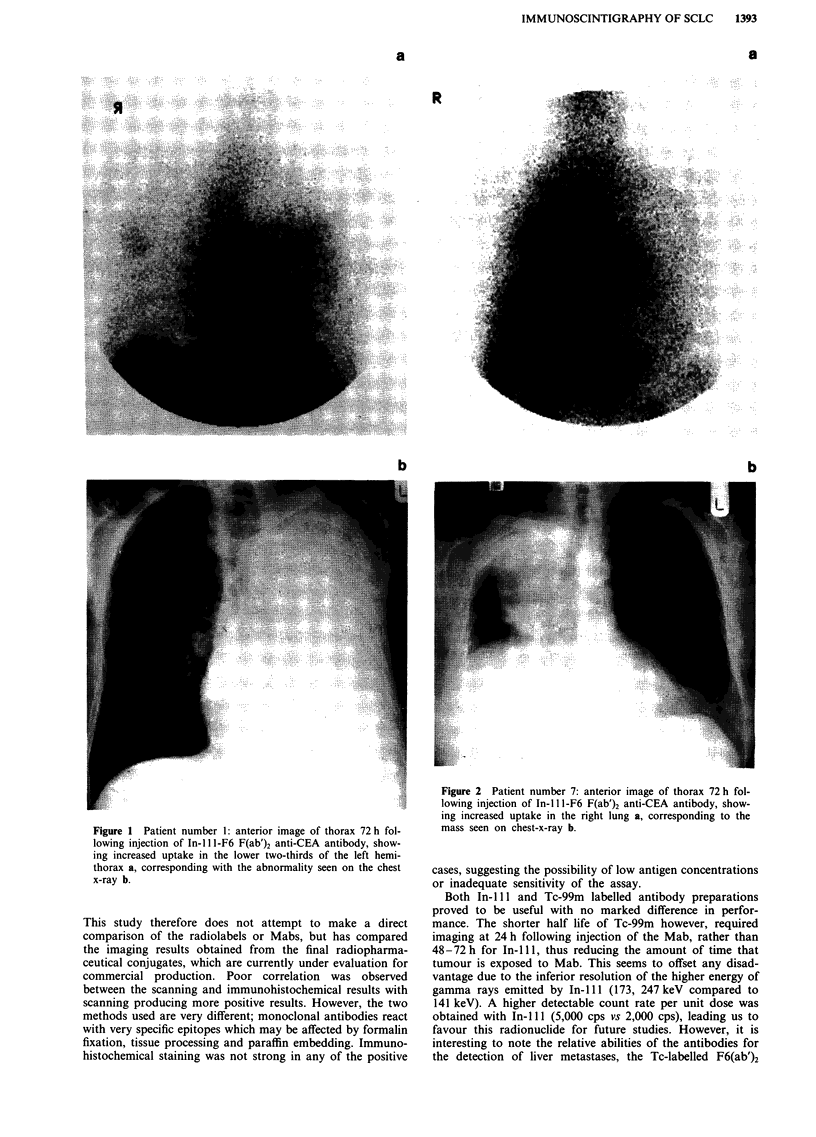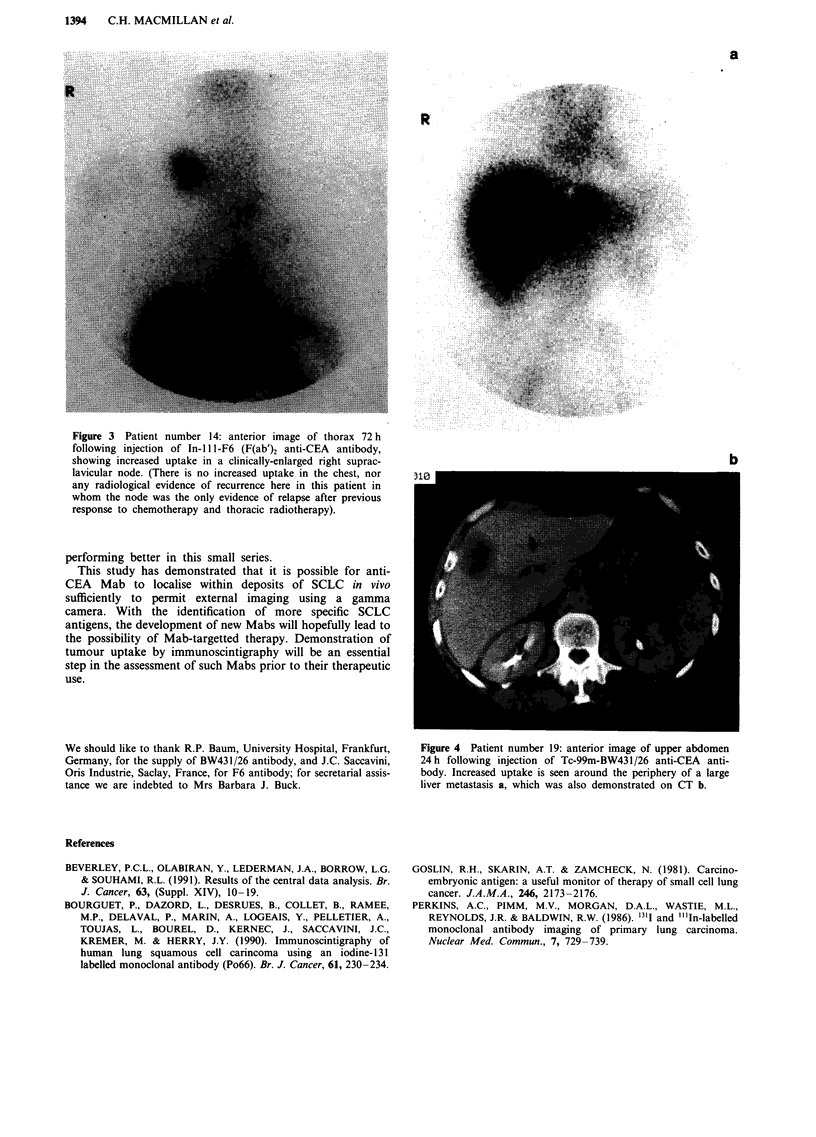# Immunoscintigraphy of small-cell lung cancer: a study using technetium and indium labelled anti-carcinoembryonic antigen monoclonal antibody preparations.

**DOI:** 10.1038/bjc.1993.257

**Published:** 1993-06

**Authors:** C. H. Macmillan, A. C. Perkins, M. L. Wastie, I. H. Leach, D. A. Morgan

**Affiliations:** Department of Clinical Oncology, General Hospital, Nottingham, UK.

## Abstract

**Images:**


Br. J. Cancer (1993), 67, 1391–1394 © Macmillan Press Ltd., 1993

### Immunoscintigraphy of small-cell lung cancer: a study using technetium and indium labelled anti-carcinoembryonic antigen monoclonal antibody preparations

C.H. Macmillan[1], A.C. Perkins[2], M.L. Wastie[3], I.H. Leach[4] & D.A.L. Morgan[1]

[1]*Department of Clinical Oncology, General Hospital, Nottingham NG1 6HA; Department of* [2]*Medical Physics and* [3]*Radiology, University Hospital, Nottingham;* [4]*Department of Histopathology, City Hospital, Nottingham, UK.*

**Summary** Immunoscintigraphy with radiolabelled anti-carcinoembryonic antigen monoclonal antibody was performed on 21 patients with active small-cell lung cancer. Patients received either In-111-labelled Mab F6 $F(ab')_2$ fragments or Tc-99m-labelled BW 431/26 intact antibody.

Tumour was imaged in 13 patients (62%). Of 38 known sites of disease, 18 sites were positively detected. Serum CEA levels were known in 19 patients and were abnormally elevated in three (tumour being detected in all three patients). Eight of 15 patients with normal serum CEA had positive imaging. Using the In-111-labelled antibody seven out of ten patients (nine out of 18 sites) gave positive scans; with the Tc-99m-labelled antibody these were obtained in nine out of 18 patients (nine out of 20 sites).

Considerable interest has developed in the identification of antigens expressed by small-cell lung cancer (SCLC) cells, and with the production of monoclonal antibodies (Mabs) which react with them. Many such Mabs are emerging. A recent serological study of 87 candidate monoclonal antibodies has resulted in the assignment of 33 Mabs to seven clusters associated with SCLC (Beverley *et al.*, 1991). If highly specific Mabs can be produced, in addition to diagnostic imaging, the possibility of targetted therapy using Mabs conjugated with toxins or therapeutic radioisotopes will arise.

Before such treatment can be considered, a Mab must be shown to selectively localise within tumours *in vivo*. If it does, then clinically detectable tumour masses should be identifiable by immunoscintigraphy. However there have been few published studies on the immunoscintigraphy of lung cancer (Perkins *et al.*, 1986; Bourguet *et al.*, 1990). If imaging is to be undertaken there is still debate concerning which radionuclide will prove to be the most useful radiolabel and which form of antibody or fragments will produce the most contrast between tumour and normal tissues.

Technetium-99m (Tc-99m) and Indium-111 (In-111) have been widely used in immunoscintigraphy. The lower gamma ray energy emitted from Tc-99m (physical half-life 6 h) gives better resolution with most imaging equipment, but the longer physical half-life of In-111 (2–8 days) permits imaging 2–3 days following administration, when the tumour-to-tissue concentration of the Mab may be more favourable.

Carcinoembryonic antigen (CEA) is an oncofoetal protein which has been reported to be expressed by up to 70% of SCLC cell lines (Goslin *et al.*, 1981). Anti-CEA Mabs are widely available, so although they are less specific for SCLC than other antibodies, they were a convenient material to use in the preliminary investigation of the immunoscintigraphy of SCLC.

The present study investigates the performance of two anti-CEA monoclonal antibodies labelled with Tc-99m or In-111 in the immunoscintigraphy of SCLC.

### Methods

Twenty-one patients were imaged; nine had newly diagnosed SCLC and 12 had relapsed after initially responding to conventional therapy. All had histologically proven disease and none had received active treatment in the month prior to imaging. Sites of involvement were determined by clinical examination, chest radiograph and supplementary radiological investigation as appropriate. The two anti-CEA Mabs used were $F(ab')_2$ fragments of antibody F6 (Oris Industrie, France) radiolabelled with 80 MBq In-111 (10 patients), and intact BW431/26 (Behringwerke, Germany) radiolabelled with 1000 MBq Tc-99m (11 patients).

Following slow intravenous injection the patients were imaged using a large field of view gamma camera (IGE 400ac) fitted with an appropriate energy collimator. Anterior and posterior images of the thorax and abdomen were recorded containing approximately 600 k counts and stored by computer in a 128 × 128 matrix. The imaging parameters are shown in Table I. Within 20 minutes of administration a 'blood pool' image was taken. This was then compared with a second scan recorded after allowing time for the Mab to localise within the tumour: 24 h in the case of Tc-99m-labelled Mab and 72 h in the case of In-111-labelled Mab. Three of the authors assessed the images, and scored the degree of tumour localisation in each patient using a subjective arbitrary scale from 0 to + + + +.

The original formalin fixed, paraffin-embedded diagnostic biopsies were available on 18 patients; the remaining three were diagnosed on cytology. These biopsies were examined immunohistochemically using a standard immunoperoxidase technique with four anti-CEA monoclonal antibodies: the two scanning antibodies (F6, BW431/26), CEA and CEA/NCA (both CRC Laboratories, Nottingham).

### Results

The results of the investigations are shown in Table II together with clinical data.

No adverse effects were observed following administration of either Mab. The images obtained from patients administered with both radiolabelled antibodies showed uptake throughout the reticulo-endothelial system with a

**Table I** Imaging parameters

| | *Tc-99m-BW431/26* | *In-111-Mab F6* |
|---|---|---|
| Molecular form | IgG1 | $F(ab')_2$ |
| Protein dose | 2 mg | 1 mg |
| Radioactive dose | 1000 MBq | 80 MBq |
| Collimator | Low energy | Medium energy |
| Photopeaks | 141 keV | 173, 247 keV |
| Imaging times | 20 min, 24 h | 20 min, 72 h |
| Approx count rates (anterior thorax) | 2000 cps | 5000 cps |

Correspondence: D.A.L. Morgan.
Received 16 July 1992; and in revised form 18 January 1993.

Table II

| Patient | | New/ recurrent | Serum CEA < 10 mcg $l^{-1}$ | Scan | | Scan sites | Disease sites |
|---|---|---|---|---|---|---|---|
| 1 | FH | R | Neg 2.1 | In | + + + | Hemithorax; liver | Hemithorax, liver; coeliac nodes. |
| 2 | RH | N | Neg 7.6 | In | + + + | R upper lobe | R upper lobe; liver |
| 3 | MB | R | Neg < 1 | In | 0 | – | Mediastinum |
| 4 | FW | R | Neg < 1 | In | 0 | – | Adrenal; mediastinum |
| 5 | JK | R | Neg < 1.5 | In | 0 | – | Adrenal; liver; neck nodes |
| 6 | EH | N | Neg 2.2 | In | + + | R middle zone | R middle zone; bone |
| 7 | MN | R | – | In | + + + + | R hilum | R hilum |
| 8 | JA | R | Neg 7.3 | In | + + + | Mediastinum; L lung | Mediastinum; L lung; bone |
| 9 | MS | N | Neg < 1 | Tc | + | Mediastinum | Mediastinum; neck node |
| 10 | AM | N | Pos 42.4 | Tc | + + + | L hilum; neck nodes | L hilum; neck nodes |
| 11 | PO | N | Neg 1.9 | Tc | 0 | – | Pleural effusion |
| 12 | NH | R | Pos 35.8 | Tc | + + + + | Liver | Pleural effusion; liver |
| 13 | WG | N | Neg 6.5 | Tc | 0 | – | R lung |
| 14 | SM | R | Neg 1.9 | In | + + + + | Neck node | Neck node |
| 15 | DE | N | Neg 9.5 | In | + + + | R lower lobe | R lower lobe; skin |
| 16 | GP | N | Neg 4.5 | Tc | + | L hilum; bone | L hilum; bone |
| 17 | BK | R | Neg < 1 | Tc | 0 | – | Hemithorax; neck node |
| 18 | NW | R | Neg 4.5 | Tc | 0 | – | Hemithorax |
| 19 | FP | R | Pos 19.6 | Tc | + + + + | R upper lobe; liver | R upper lobe; liver |
| 20 | DL | R | – | Tc | + + + | R upper zone | R upper zone; neck node |
| 21 | AMN | N | – | Tc | 0 | – | Mediastinum |

high level of activity in the liver. This pattern of uptake was evident from the early blood pool images and persisted in the later views. The early blood pool views of the thorax demonstrated activity in the heart which was reduced in the later views. Positive uptake was observed at tumour sites in all three patients known to have elevated levels of serum CEA, and also in nine who showed no abnormality in serum marker level. One other patient whose serum CEA was unknown also had a positive scan, making a total of 13 patients with positive imaging (62%). In ten of these, the degree of tumour localisation was assessed as strongly positive (+ + + or + + + +). Within the group of 21 patients, 38 separate sites of bulk diseases were known, and 18 of these were positively imaged (47%). Four examples of positive scans are illustrated (Figures 1–4).

Of ten patients studied with In-111 labelled antibody, seven were positive (9/18 sites) as were 6/11 receiving Tc-99m (9/20 sites). A total of five patients had liver metastases. Liver metastases were detected in 2/2 patients studied with Tc-99m-Mab whereas use of the In-111-F(ab')$_2$ antibody failed to detect liver metastases in three patients.

Immunohistochemical examination of the 18 histological specimens using the panel of four anti-CEA Mabs gave positive staining in five; four of these were positive to all four Mabs and the other reacted to the two Mabs not used for scanning, but not the the scanning Mabs. Of the five positive on immunohistochemistry, three had given positive scans; conversely there were eight assessed histochemically on whom positive scans were obtained but who showed no immunoreactivity. Thus, no correlation was seen between uptake as shown by immunoscintigraphy and that of immunohistochemistry.

### Discussion

In SCLC, only patients achieving complete response to conventional treatment have stood any chance of cure, but even in this group resistant micrometastases lead to relapse and ultimate death for the great majority. Targetted therapy with Mabs reacting with SCLC tumour antigens might be a way of eradicating such micrometastases, and thereby increasing cure rates. Before such treatments can be considered, uptake of Mab by the tumour must be demonstrated. Immunoscintigraphy provides a useful means of showing such uptake *in vivo*. There are, however, little published data on the localisation of SCLC by immunoscintigraphy. We have previously demonstrated successful imaging of various histological types of lung cancer (SCLC included) using an anti-72000 dalton glyoprotein associated antibody (Perkins *et al.*, 1986). Our present study has demonstrated that detectable uptake by SCLC occurs *in vivo* in more than half the patients studied using anti-CEA Mabs which are not specific to SCLC.

The relationship of serum CEA with outcome of the scan is surprising. All three patients with elevated serum CEA had positive imaging, but it is interesting to note that eight of 15 patients with normal serum CEA still had positive scans. Although Goslin *et al.* (1986) reported 70% of patients with SCLC to have elevated serum CEA, we saw only three patients with raised levels out of 18 assessable (17%). It is pertinent to note that Goslin *et al.* used a serum assay with an upper limit of normal for serum CEA of 2.5 mcg.$l^{-1}$ whereas we used an assay with a higher value of 10 mcg.$l^{-1}$. Six patients had serum CEA levels between 2.5–10 mcg.$l^{-1}$, and of these, four had positive imaging. It is of course possible that false-positive uptake visualised on imaging is due in part to non-specific localisation in inflammatory tissue. This consideration would apply to all studies using radiolabelled antibodies and could only be clarified by using a non-specific immunoglobulin of the same isotype used for imaging. It was not possible to perform this study in the present series of patients.

It is important to emphasise that the anti-CEA antibody preparations used in the present study were different, with intact BW 431/26 antibody radiolabelled with Tc-99m and F6(ab')$_2$ fragments radiolabelled with In-111. Factors such as antibody fragmentation, affinity constant and choice of radiolabel will all affect the performance of the preparations.

**Figure 1** Patient number 1: anterior image of thorax 72 h following injection of In-111-F6 $F(ab')_2$ anti-CEA antibody, showing increased uptake in the lower two-thirds of the left hemithorax **a**, corresponding with the abnormality seen on the chest x-ray **b**.

**Figure 2** Patient number 7: anterior image of thorax 72 h following injection of In-111-F6 $F(ab')_2$ anti-CEA antibody, showing increased uptake in the right lung **a**, corresponding to the mass seen on chest-x-ray **b**.

This study therefore does not attempt to make a direct comparison of the radiolabels or Mabs, but has compared the imaging results obtained from the final radiopharmaceutical conjugates, which are currently under evaluation for commercial production. Poor correlation was observed between the scanning and immunohistochemical results with scanning producing more positive results. However, the two methods used are very different; monoclonal antibodies react with very specific epitopes which may be affected by formalin fixation, tissue processing and paraffin embedding. Immunohistochemical staining was not strong in any of the positive cases, suggesting the possibility of low antigen concentrations or inadequate sensitivity of the assay.

Both In-111 and Tc-99m labelled antibody preparations proved to be useful with no marked difference in performance. The shorter half life of Tc-99m however, required imaging at 24 h following injection of the Mab, rather than 48–72 h for In-111, thus reducing the amount of time that tumour is exposed to Mab. This seems to offset any disadvantage due to the inferior resolution of the higher energy of gamma rays emitted by In-111 (173, 247 keV compared to 141 keV). A higher detectable count rate per unit dose was obtained with In-111 (5,000 cps *vs* 2,000 cps), leading us to favour this radionuclide for future studies. However, it is interesting to note the relative abilities of the antibodies for the detection of liver metastases, the Tc-labelled $F6(ab')_2$

**Figure 3** Patient number 14: anterior image of thorax 72 h following injection of In-111-F6 (F(ab')$_2$ anti-CEA antibody, showing increased uptake in a clinically-enlarged right supraclavicular node. (There is no increased uptake in the chest, nor any radiological evidence of recurrence here in this patient in whom the node was the only evidence of relapse after previous response to chemotherapy and thoracic radiotherapy).

**Figure 4** Patient number 19: anterior image of upper abdomen 24 h following injection of Tc-99m-BW431/26 anti-CEA antibody. Increased uptake is seen around the periphery of a large liver metastasis **a**, which was also demonstrated on CT **b**.

performing better in this small series.

This study has demonstrated that it is possible for anti-CEA Mab to localise within deposits of SCLC *in vivo* sufficiently to permit external imaging using a gamma camera. With the identification of more specific SCLC antigens, the development of new Mabs will hopefully lead to the possibility of Mab-targetted therapy. Demonstration of tumour uptake by immunoscintigraphy will be an essential step in the assessment of such Mabs prior to their therapeutic use.

We should like to thank R.P. Baum, University Hospital, Frankfurt, Germany, for the supply of BW431/26 antibody, and J.C. Saccavini, Oris Industrie, Saclay, France, for F6 antibody; for secretarial assistance we are indebted to Mrs Barbara J. Buck.

### References

BEVERLEY, P.C.L., OLABIRAN, Y., LEDERMAN, J.A., BORROW, L.G. & SOUHAMI, R.L. (1991). Results of the central data analysis. *Br. J. Cancer*, **63**, (Suppl. XIV), 10-19.

BOURGUET, P., DAZORD, L., DESRUES, B., COLLET, B., RAMEE, M.P., DELAVAL, P., MARIN, A., LOGEAIS, Y., PELLETIER, A., TOUJAS, L., BOUREL, D., KERNEC, J., SACCAVINI, J.C., KREMER, M. & HERRY, J.Y. (1990). Immunoscintigraphy of human lung squamous cell carincoma using an iodine-131 labelled monoclonal antibody (Po66). *Br. J. Cancer*, **61**, 230-234.

GOSLIN, R.H., SKARIN, A.T. & ZAMCHECK, N. (1981). Carcinoembryonic antigen: a useful monitor of therapy of small cell lung cancer. *J.A.M.A.*, **246**, 2173-2176.

PERKINS, A.C., PIMM, M.V., MORGAN, D.A.L., WASTIE, M.L., REYNOLDS, J.R. & BALDWIN, R.W. (1986). $^{131}$I and $^{111}$In-labelled monoclonal antibody imaging of primary lung carcinoma. *Nuclear Med. Commun.*, **7**, 729-739.